# Comparative Immune Phenotypic Analysis of Cutaneous Squamous Cell Carcinoma and Intraepidermal Carcinoma in Immune-Competent Individuals: Proportional Representation of CD8^+^ T-Cells but Not FoxP3^+^ Regulatory T-Cells Is Associated with Disease Stage

**DOI:** 10.1371/journal.pone.0110928

**Published:** 2014-10-23

**Authors:** Andrew Freeman, Jennifer A. Bridge, Pirashanthini Maruthayanar, Nana H. Overgaard, Ji-Won Jung, Fiona Simpson, Tarl W. Prow, H. Peter Soyer, Ian H. Frazer, Michael Freeman, James W. Wells

**Affiliations:** 1 The Skin Centre, Pindara Private Hospital, Gold Coast, Australia; 2 Dermatology Research Centre, The University of Queensland, School of Medicine, Translational Research Institute, Brisbane, Australia; 3 The University of Queensland Diamantina Institute, University of Queensland, Translational Research Institute, Brisbane, Australia; University of Palermo, Italy

## Abstract

Squamous Cell Carcinoma (SCC) is a type of non-melanoma skin cancer prevalent in immune-suppressed transplant recipients and older individuals with a history of chronic sun-exposure. SCC itself is believed to be a late-stage manifestation that can develop from premalignant lesions including Intraepidermal Carcinoma (IEC). Notably, while SCC regression is rare, IEC typically regresses in response to immune modifying topical treatments, however the underlying immunological reasons for these differential responses remain unclear. This study aimed to define whether IEC and SCC are associated with distinct immune profiles. We investigated the immune cell infiltrate of photo-damaged skin, IEC, and SCC tissue using 10-colour flow cytometry following fresh lesion digest. We found that IEC lesions contain higher percentages of CD3^+^ T-cells than photo-damaged skin, however, the abundance of CD3^−^CD56^+^ Natural Killer (NK) cells, CD11c^+^HLA-DR^+^ conventional Dendritic Cells (cDC), BDCA-2^+^HLA-DR^+^ plasmacytoid DC (pDC), FoxP3^+^ Regulatory T-cells (T-reg), Vα24^+^Vβ11^+^ invariant NKT-cells, and γδ Tcells did not alter with disease stage. Within the total T-cell population, high percentages of CD4^+^ T-cells were associated with SCC, yet CD8^+^ T-cells were less abundant in SCC compared with IEC. Our study demonstrates that while IEC lesions contain a higher proportion of T-cells than SCC lesions in general, SCC lesions specifically display a lower abundance of CD8^+^ T-cells than IEC. We propose that differences in CD8^+^ T-cell abundance contribute critically to the different capacity of SCC and IEC to regress in response to immune modifying topical treatments. Our study also suggests that a high ratio of CD4^+^ T-cells to CD8^+^ T-cells may be a immunological diagnostic indicator of late-stage SCC development in immune-competent patients.

## Introduction

Cutaneous Squamous Cell Carcinoma (SCC) typically presents in immune competent patients over the age of 50. Years of sun exposure lead to DNA damage and mutations in the tumour suppressor protein p53; the same p53 mutations found in >90% of cutaneous SCCs are also found in precancerous lesions like actinic keratosis (AK) [1]. AKs and invasive SCC are generally considered to be at the early and late ends of the same disease spectrum [2], with Intraepidermal Carcinoma (IEC), also known as SCC *in situ*, lying somewhere in between. While SCC is rarely fatal if treated early, the disease is especially prone to recurrence as a result of irreversible sun-damage to the skin, and patients endure significant disfigurement, both from the disease and from its management.

The overwhelming majority of precancerous AK and IEC lesions in immune competent patients eventually regress, with as little as 1–10% estimated to progress to invasive SCC [3], [4]. In contrast, AKs are known to occur more frequently, develop sooner, and progress more rapidly to SCC in long-term immune-suppressed patients [5]. Particularly at risk are organ transplant recipients, who suffer a 10–100 fold increased risk of developing SCC when compared with the general population [6]. Recent clinical studies have reported that the complete regression of precancerous lesions can be induced in some patients through the topical application of the immune-modulators Imiquimod [7], [8] and Diclofenac [9]. SCC, by contrast, displays a low clinical response rate to Imiquimod, and Imiquimod is not recommended for the therapy of invasive SCC [10]. Whether this lack of responsiveness of is due to differences in the underlying immunology, the pharmacological depth of penetration of topical compounds, differences in blood and lymph vasculature or the sheer number of tumour cells making an immune response to a larger tumour more difficult, is currently unknown.

In this study, we investigated the immune cell infiltrate of photo-damaged skin, IEC, and SCC tissue using 10-colour flow cytometry to determine whether IEC and SCC display fundamental differences in immune cell composition. Our findings demonstrate that significant differences exist within the T-cell compartment in IEC and SCC lesions.

## Methods

### Patient samples

All patient samples were collected from outpatients at The Skin Centre, Pindara Private Hospital, Gold Coast. The study was approved by The University of Queensland Medical Research Ethics Committee. Written, informed consent was obtained from all patients prior to participation, and the study was performed with strict adherence to the Declaration of Helsinki Ethical Principles.

A total of 25 patients with a history of IEC or SCC formation (typically >5 lesions in the previous 10 years) were included in this study (11 females and 14 males). The median age was 68.0 (54 to 88) for females and 75 (62 to 88) for males, and the vast majority of lesions were derived from moderately or severely sun-damaged sites on the face, upper arm, or calf. Patient and sample characteristics are summarised in Table 1. In total, 6 photo-damaged skin samples, 9 IEC lesions, and 13 SCC lesions were analysed. Following lesion removal, fresh, excess tissue not required for diagnostic purposes was de-identified prior to analysis at The University of Queensland Diamantina Institute, Translational Research Institute, Brisbane. Specimen pathology including H&E staining was performed by Helix Pathology (Southport, Queensland) and diagnosis correlated to sample analysis post-hoc by the treating clinician. Analysis data were not made available to the treating clinician prior to correlation. Specimens were either deep shave excisions or elliptical excisions; SCC lesions included keratoacanthoma subtypes [11]. Photo-damaged skin was derived from sun-exposed ellipses of excision specimens and was 1 cm from the tumours.

**Table 1:** Patient and sample characteristics.

Skin Type	Specimen	Area	Age	Sex	History of skin cancers (previous 10 yrs)	Solar Damage
**II**	Well diff. SCC	Scalp/forehead	78	M	<10 SCC, Mel	Mild
**I**	KA	Face	87	M	>20 SCC, <10 BCC^a^	Severe
**I**	KA	Back	66	F	>85 SCC, <10 BCC, Mel	Mod-severe
	Well diff. SCC	Face	88	M	<10 SCC	Moderate
	SCC	-	69	F	<5 SCC, Mel	Mild
**I**	IEC	Right Lateral Calf	80	M	17 SCC, <5 BCC, Mel^c^	Moderate
**I**	IEC	Left Posterior Calf	87	M	>20 SCC, <10 BCC^a^	Severe
**II**	IEC	Anterior Chest	71	M	1 BCC, >5 IECs, KA	Mild
**II**	IEC	Left dorsal forearm	76	M	>10 BCCs, >10 IECs, 1 SCC	Moderate
**II**	IEC	Right hand lateral	54	F	>20 IECs, >20 SCCs, 3 BCCs^b^	Severe
	IEC	Face	68	F	24 SCC	Moderate
**I**	IEC	Left Lateral Calf	85	F	>10 SCC, <5 BCC	Mod-severe
	IEC	Left Lateral Calf	88	F	>5 SCC, <10 BCC	Moderate
**II**	SCC	Hand	54	F	>20 IECs, >20 SCCs, 3 BCCs^b^	Severe
**I**	SCC	Right dorsal arm	68	F	3 SCCs, 3 BCCs, 3 IECs, Mel	Moderate
**I**	SCC	Right Calf	80	M	17 SCC, <5 BCC, Mel^c^	Moderate
**II**	Well diff. SCC	Right Medial Calf	64	M	>15 SCC	Severe
**II**	SCC	Left Elbow	72	M	<5 SCC, <10 BCC	Moderate
	Skin	Face	54	F	<5 BCC	Moderate
	Skin	Left upper arm	69	M	5 SCC, 8 BCC	Mod-severe
	Skin	Left upper arm	69	F	Mel	Mild
	Skin	Left shoulder	77	M	<5 BCC	Mod-severe
**II**	Skin	Right upper arm	77	F	<5 SCC, 47 BCC	Severe
**II**	Skin	Face	87	M	<5 SCC, 7 BCC	Moderate
	IEC	Chest	62	M	1 BCC, Mel	Severe
**II**	Well diff. SCC	Right Lateral Calf	69	M	10 SCC, 7 BCC, Mel	Moderate
**I**	Well diff. SCC	Chest	66	F	>70 SCC, <10 BCC	Mod-severe
**I**	KA	Left Posterior Calf	74	M	<10 SCC, 4 BCC, Mel	Moderate

Skin types are indicated where recorded. Well diff: Well differentiated, IEC: Intraepidermal Carcinoma, SCC: Squamous Cell Carcinoma, BCC: Basal Cell Carcinoma, KA: Keratoacanthoma, Mel: Melanoma, Mod: Moderate.

a,b,c these samples were harvested from the same patents but several months apart.

### Antibodies and reagents

The following antibodies and reagents were used for sample analysis by flow cytometry: Human Fc receptor binding inhibitor (eBioscience), Anti-CD45 PE-Cy7 (HI30; eBioscience), anti-CD11b PE (M1/70; BD Biosciences), anti-CD56 BV421 (HCD56; Biolegend), anti-CD3 PerCp-Cy5.5 (OKT3, eBioscience), anti-CD4 Alexa Fluor 700 (OKT4; eBioscience), anti-CD8a APC-Cy7 (HIT8a; Biolegend), anti-Vα24 PE (C15; Beckman Coulter), anti-Vβ11 FITC (C21; Beckman Coulter), anti-BDCA-2 APC (201A; Biolegend), anti-TCRγδ PE-CF594 (B1; BD Biosciences), anti-CD11c BV421 (3.9; Biolegend), anti-HLA-DR PE-CF594 (G46-6; BD Biosciences), anti-Cytokeratin 10 biotin (DE-K10; Abcam), anti-Cytokeratin 14 FITC (LL002; Abcam), Streptavidin-APC-Cy7 (BD Biosciences), and the anti-Human FoxP3 Staining Set (236A/E7; eBioscience). Rat IgG2a,κ Isotype Control APC (R35-95; BD Biosciences) was used as an isotype control for intracellular FoxP3 staining. Dead cells were excluded using the LIVE/DEAD Fixable Aqua Dead Cell Stain Kit (Life Technologies, New York, U.S.) according to the manufacturers’ instructions, and anti-CD45 was used to differential immune cells from non-immune cells.

### Immune phenotyping

Immune phenotyping was performed on fresh tissue within 2 hours of harvest. All samples were collected in sterile saline and kept on ice during transit. For cell isolation, tissues were cut into small fragments with scalpel blades, before being digested in pre-heated RPMI/2% FBS/3 mg/mL collagenase D/5 µg/mL DNase 1 (both from Roche Applied Science, Victoria, Australia) for 90 mins at 37°C. Samples were vortexed every 30 minutes to facilitate digestion. Cells were then passed through a 70 µm cell strainer (BD Biosciences, New Jersey, USA) and subsequently through a 40 µm cell strainer (BD Biosciences). Once isolated, cells were resuspended in PBS/0.5% FBS and divided across two panels for staining. To block non-specific antibody staining, cells were incubated with 20 µl Human Fc receptor binding inhibitor for 20 minutes on ice. Cells were then stained with monoclonal antibodies to elucidate intracellular Keratin 10 and Keratin 14 expression in viable CD45^−^ populations, and surface stained to elucidate the following immune populations in viable CD45^+^ populations: CD3^+^ T-cells, CD3^−^CD56^+^ NK cells, CD11c^+^HLA-DR^+^ conventional DC (cDC), and BDCA-2^+^HLA-DR^+^ pDC. T-cell subsets were further elucidated based on CD3^+^CD4^+^ for CD4^+^ T-cells, CD3^+^CD8^+^ for CD8^+^ T-cells, CD3^+^TCRγδ^+^ for gamma/delta T-cells, and CD3^+^Vα24^+^Vβ11^+^ invariant Natural Killer T-cells (iNKT). Intracellular staining for Foxp3 to elucidate FoxP3^+^ T-regs was performed using the anti-human FoxP3 staining kit from ebioscience (ebioscience) according to the manufacturers’ instructions, and isotype-control staining was performed in parallel. The percentage of FoxP3^+^ cells was determined after gating on CD3^+^CD4^+^ cells. Antibodies for surface staining were added for 30 minutes on ice, and antibodies for intracellular staining were added overnight at 4°C. Analysis was performed by flow cytometry using a Gallios Flow Cytometer (Beckman Coulter) and data analysed using Kaluza software (version 1.2, Beckman Coulter).

### Statistical analysis

All statistical analysis was carried out using GraphPad Prism version 6.02 for Windows GraphPad Software, San Diego, CA, USA. Whiskers in Box and Whisker plots represent the 10–90 percentile with outliers plotted as individual dots. Statistical comparisons of ranked values were conducted using a Kruskal-Wallis test with Dunn’s post-hoc test for multiple comparisons, followed by Bonferroni’s correction to adjust the threshold for the number of markers tested. *P* values of *p*<0.05 (*) were considered significant. *p*<0.01 (**) and *p*<0.005 (***) are indicated.

## Results

### Epithelial and immune cells present within precancerous IEC and cancerous SCC lesions are readily detectable by flow cytometry

**Figure 1:**
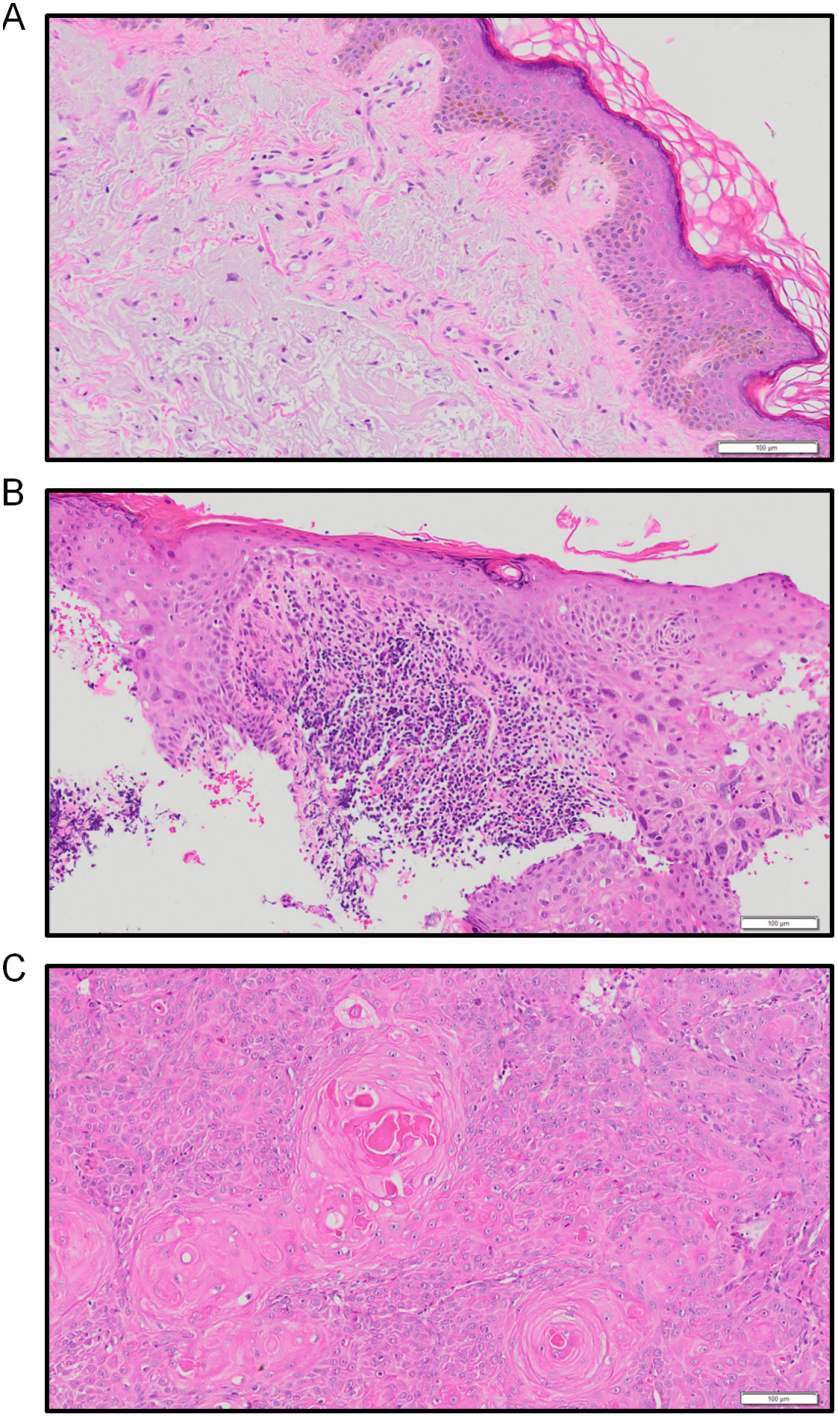
Representative photomicrographs of each of the three analysis groups. (A) Moderate to severe photo-damage in the upper dermis, (B) IEC, and (C) moderately differentiated SCC. Images presented are H&E stains, scale bar = 100 µm.

**Figure 2:**
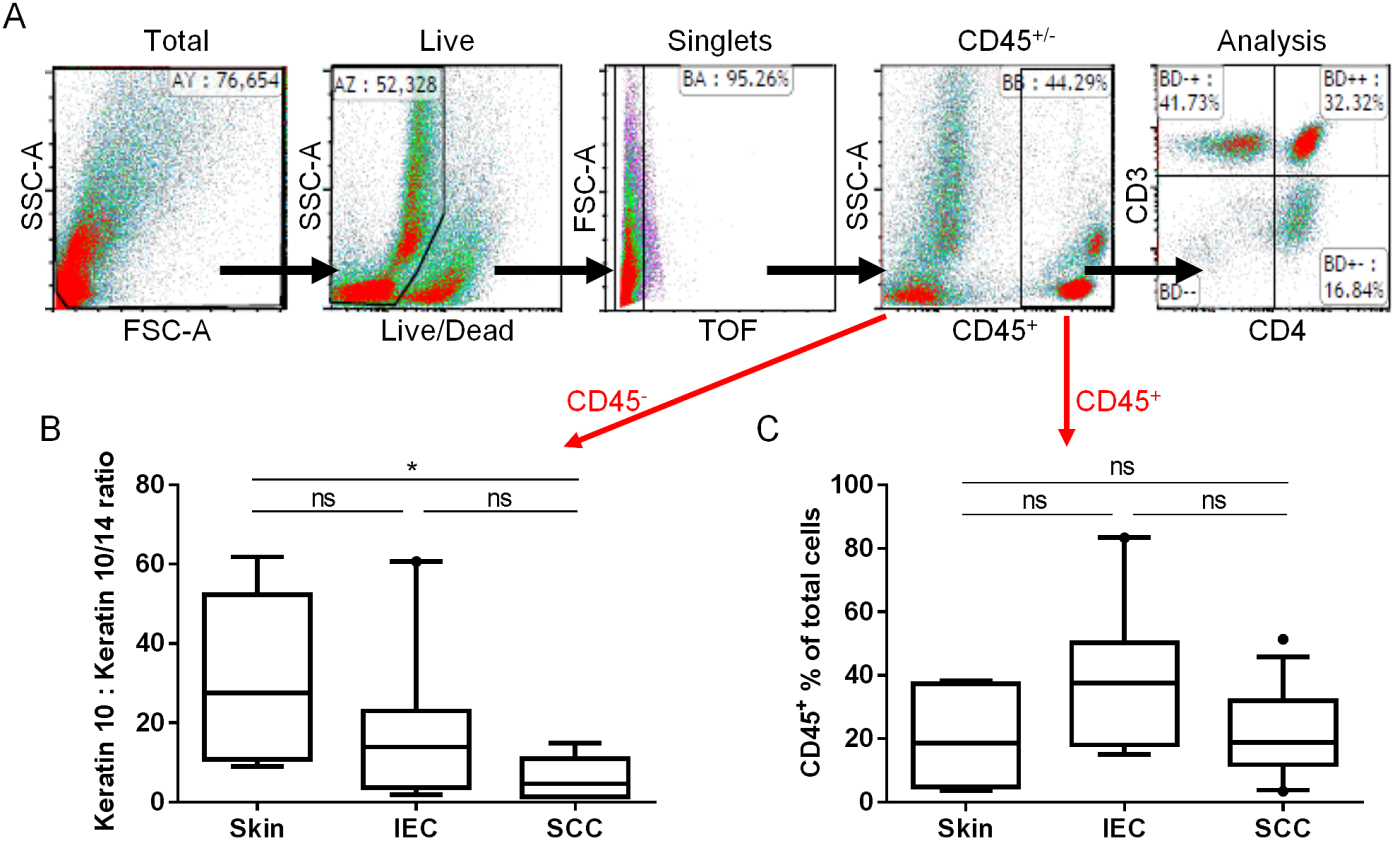
Gating strategy for analysis of immune cell populations in tumour lesions by flow cytometry. Age-matched sun-exposed skin or tumour lesions were excised and digested as outlined in *Materials & Methods*. Samples were stained with antibody and analysed by flow cytometry using the gating strategy shown in (A). (B) Ratio of Keratin 10^+^ Keratin 14^−^ cells (suprabasal keratinocytes) to Keratin 10^+^ Keratin 14^+^ cells (basal keratinocytes) in the live CD45^−^ population as determined by intracellular staining. (C) Comparison of viable immune cell extraction showing similar extraction efficacy between sample types. Live CD45^+^ cells as a percentage of the total live cell population is shown (skin; n = 6, IEC; n = 9, SCC; n = 13). ns: not significant. SSC-A: Side Scatter-Area, FSC-A: Forward Scatter-Area, TOF: Time of flight.

The assessment of immune infiltrates into human skin lesions is typically performed using immunohistochemistry or immunofluorescence. While these techniques result in excellent spatial information, it is rarely possible to assess the multiple intracellular and surface markers necessary to delineate multiple subsets of immune cells at the same time. In order to accurately assess the immune microenviroment within histopathologically-defined stages of SCC disease progression (Fig. 1), we used highly sensitive 10-colour flow cytometry. Fresh lesions were digested with collagenase/DNase and stained with monoclonal antibodies to intracellular and cell surface markers for analysis (Fig. 2). To ensure accurate data interpretation, we included the use of LIVE/DEAD Cell Stain to discriminate live and dead cells (live cells exclude the stain), gated on single cells (singlets), and used the expression of the surface marker CD45 to clearly distinguish CD45^−^ epithelial cells from CD45^+^ haematopoietic (immune) cells (Fig. 2A). Within the epithelial cell population, the proportion of suprabasal (Keratin 10^+^ Keratin 14^−^) keratinocytes to basal (Keratin 10^+^ Keratin 14^+^) keratinocytes significantly decreased in SCC compared with photo-damaged skin (*p*<0.05; Fig. 2B), which is in line with the increased expression of Keratin 14 as a differential marker of SCC [12], [13]. Notably, in spite of differing architectures between photo-damaged skin, IEC, and SCC, the overall proportional representation of CD45^+^ immune cells within sample types was similar (Fig. 2C). Together, these data indicate that epithelial and immune cells are effectively delineated in IEC and SCC lesions using a flow-based analysis approach.

### Analysis of immune cell populations within IEC and SCC lesions

**Figure 3:**
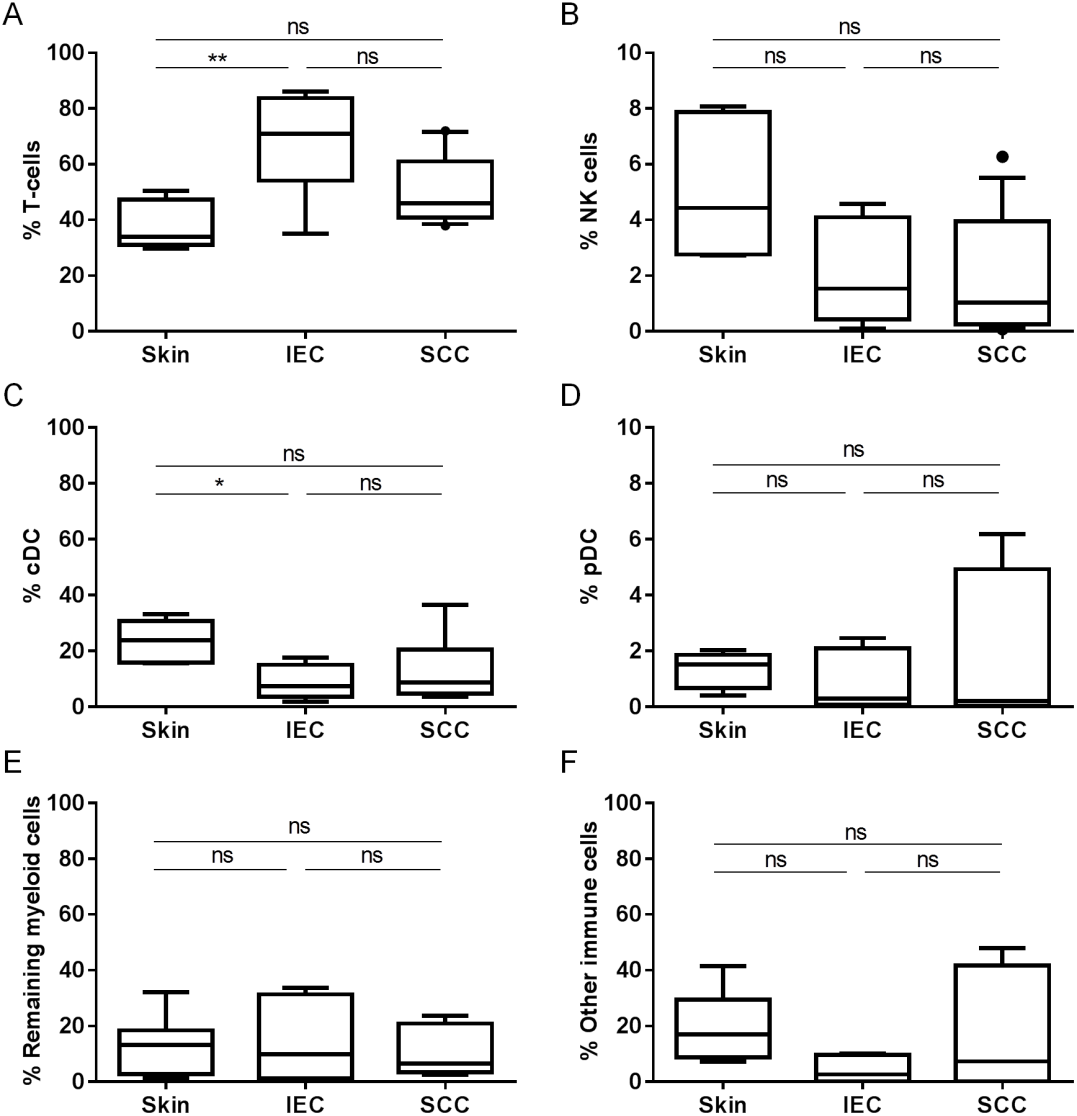
T-cells, NK cells, and DC detected in photo-damaged skin and lesional skin of patients with IEC and SCC. Fresh biopsies were digested to release cells and subsequently stained with antibodies to determine the representation of immune subpopulations by flow cytometry. (A) CD3^+^ T-cells (skin; n = 6, IEC; n = 9, SCC; n = 13), (B) CD3^−^CD56^+^ NK cells (skin; n = 6, IEC; n = 8, SCC; n = 13), (C) CD11c^+^HLA-DR^+^ cDC (includes both CD11b^hi^ and CD11b^−/lo^ populations, (skin; n = 6, IEC; n = 7, SCC; n = 7), (D) BDCA-2^+^HLA-DR^+^ pDC (skin; n = 6, IEC; n = 7, SCC; n = 7), (E) remaining CD11b^+^ myeloid cell populations (skin; n = 6, IEC; n = 7, SCC; n = 7), and (F) remaining immune cells (skin; n = 6, IEC; n = 6, SCC; n = 7). All data displayed are % of total CD45^+^ immune cell population. ns: not significant.

In order to determine the immune phenotype of IEC and SCC lesions, we compared the proportion of T-cells, Natural Killer (NK) cells, conventional Dendritic Cells (cDC) and plasmacytoid DC (pDC) in biopsied lesions with photo-damaged skin derived from 6 age-matched donors (Fig. 3). As shown in Figure 3A, CD3^+^ T-cells were the most abundant immune population present in the large majority of samples. The percentage of T-cells was significantly higher in IEC as compared with photo-damaged skin (*p*<0.01). When we evaluated the percentages of CD3^−^CD56^+^ NK cells present in the specimens, we found comparable NK percentages between IEC and SCC. While both IEC and SCC appeared to show decreased NK cell abundance when compared with photo-damaged skin, statistical significance was not reached (*P* = 0.06; Fig. 3B). IEC lesions, but not SCC lesions, also showed decreased cDC abundance when compared with photo-damaged skin (*p*<0.05; Fig. 3C). However, proportions of BDCA-2^+^HLA-DR^+^ pDC (Fig. 3D) and remaining (undefined) CD11b^+^ myeloid cells (Fig. 3E) were comparable between IEC, SCC, and photo-damaged skin. Finally, on average, approximately 10–20% of immune cells in photo-damaged skin and SCC, and approximately 5% of immune cells in IEC, could not be identified using our panel of lineage markers (Fig. 3F). In conclusion, multiple immune cell types were simultaneously identified within each lesion; however the data indicate T-cell predominance; particularly within IEC lesions.

### IEC and SCC lesions display distinct patterns of CD4^+^ and CD8^+^ T-cell infiltration

**Figure 4:**
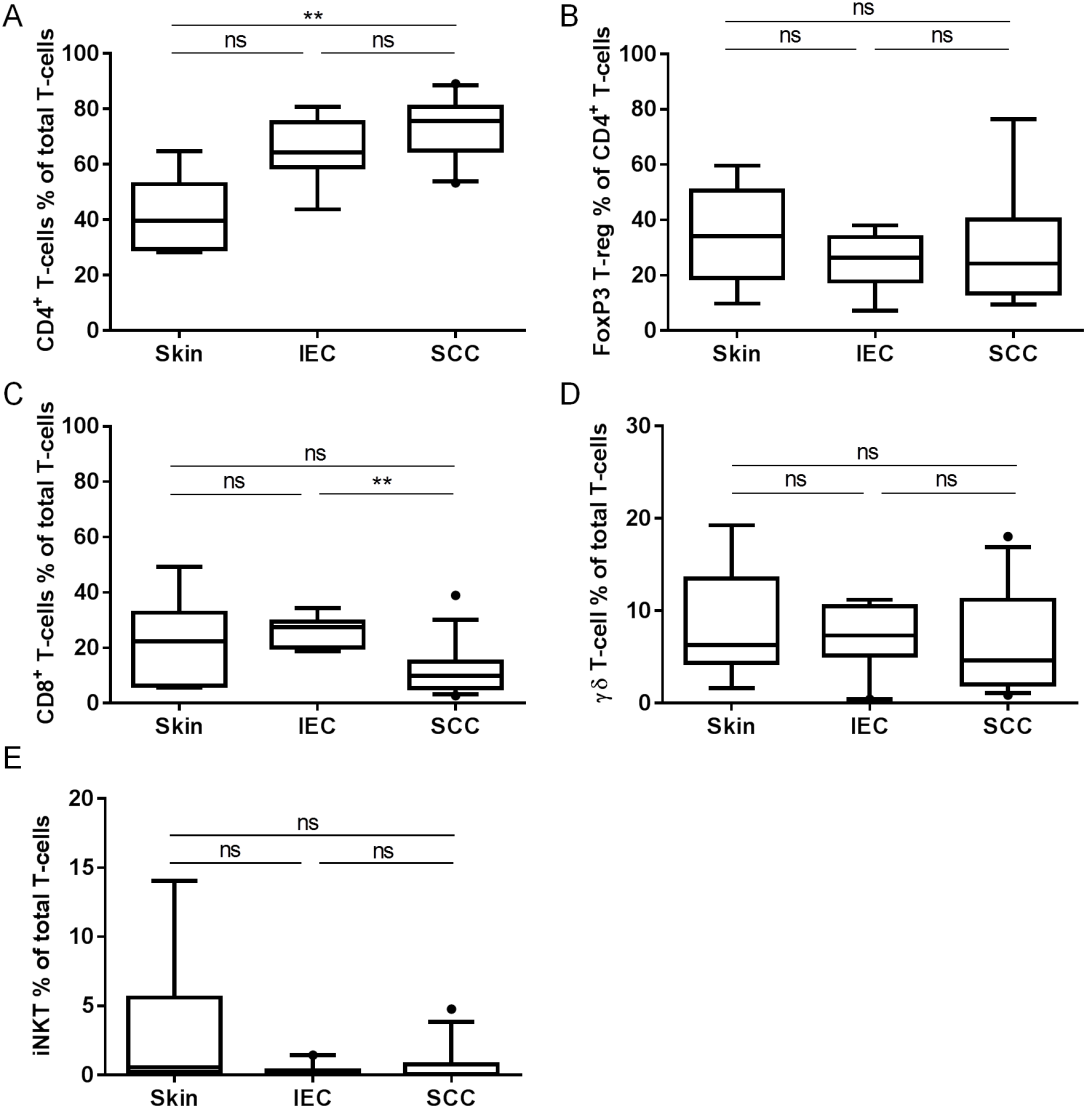
IEC and SCC lesions display distinct patterns of CD4^+^ and CD8^+^ T-cell infiltration. Fresh biopsies were digested to release cells and subsequently stained with antibodies to determine the representation of T-cell subpopulations by flow cytometry; (A) CD4^+^ T-cells (skin; n = 6, IEC; n = 9, SCC; n = 10), (B) FoxP3^+^ T-regs (skin; n = 6, IEC; n = 7, SCC; n = 8), (C) CD8^+^ T-cells (skin; n = 6, IEC; n = 8, SCC; n = 13), (D) TCRγδ^+^ T-cells (skin; n = 6, IEC; n = 9, SCC; n = 11), and (E) Vα24^+^Vβ11^+^ invariant Natural Killer T-cells (iNKT; (skin; n = 6, IEC; n = 9, SCC; n = 12). All data displayed are % of total CD3^+^ T-cells with the exception of (B) which represents the % of total CD3^+^CD4^+^ T-cells. ns: not significant.

Having observed a large T-cell presence within IEC and SCC lesions, we sought to determine the identity of the T-cell populations and whether there were differences in the abundance of specific T-cell subpopulations between lesions. Using the same samples shown in Figure 3, and following the gating of CD45^+^CD3^+^ T-cells, 5 subsets were distinguished based on the expression of CD4 (CD4^+^ T-cells), CD4 and FoxP3 (Regulatory T-cells; T-regs), CD8 (CD8^+^ T-cells), TCRγ/δ (γ/δ T-cells), and Vα24/Vβ11 (invariant NK T-cells; iNKT). As shown in Figure 4A, only SCC showed significantly increased CD4^+^ T-cell percentages when compared with photo-damaged skin (*p*<0.01), although the CD4^+^ T-cell percentages between IEC and SCC were comparable (*p* = 0.65). Interestingly, the percentage of FoxP3^+^T-reg within the CD4^+^ T-cell population did not change significantly between samples (Fig. 4B), indicating that the proportion of FoxP3^+^T-regs to the total CD4^+^ T-cell pool was not associated with disease progression. Notably however, the percentage of CD8^+^ T-cells was significantly decreased in SCC as compared to IEC (*p*<0.01; Fig. 4C), although significance was not reached when SCC was compared to photo-damaged skin (*p* = 0.26). The percentages of γ/δ T-cells (Fig. 4D) and iNKT cells (Fig. 4E) were comparable between IEC, SCC, and photo-damaged skin. The data suggest that SCC are associated with a higher percentage of CD4^+^ T-cells when compared to photo-damaged skin, although SCC may also be associated with a lower percentage of CD8^+^ T-cells when compared to IEC.

### SCC displays a higher CD4^+^ T-cell to CD8^+^ T-cell ratio than IEC or photo-damaged skin

**Figure 5:**
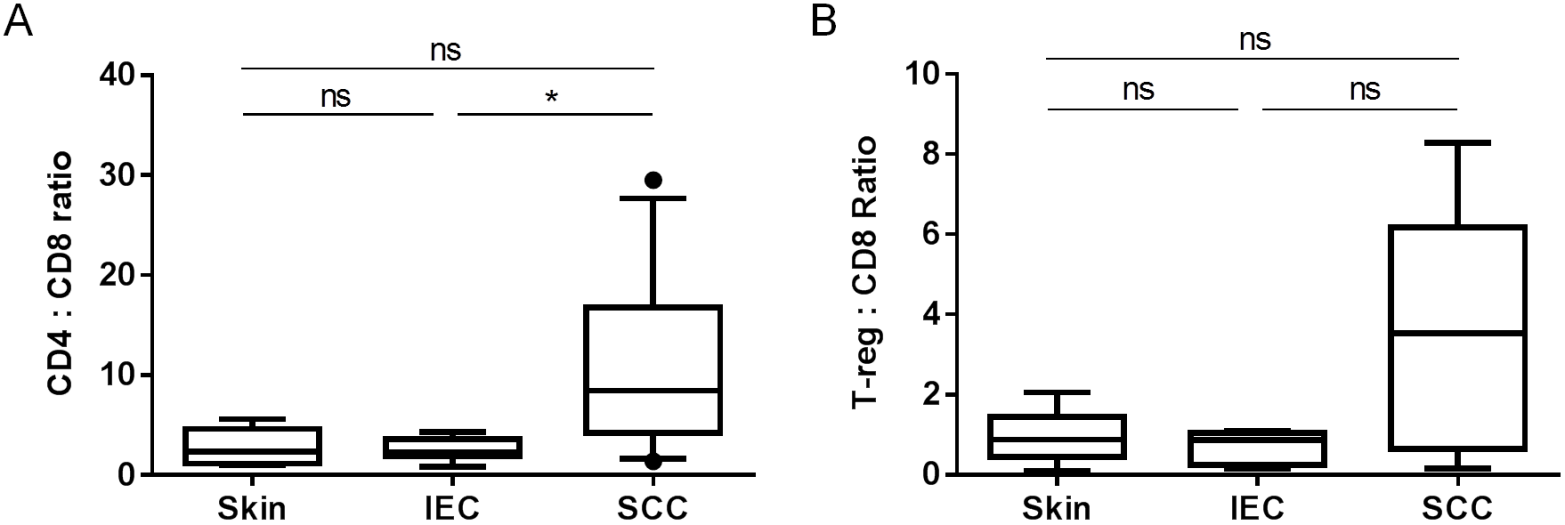
SCC lesions display both an increased CD4/CD8 ratio and an increased T-reg/CD8 ratio when compared with IEC lesions. Absolute CD3^+^CD4^+^ T-cell, CD3^+^CD4^+^FoxP3^+^ T-cell, and CD3^+^CD8^+^ T-cell numbers within each lesion were determined by flow cytometry and used to determine the proportion of (A) CD3^+^CD4^+^ T-cells to CD3^+^CD8^+^ T-cells (skin; n = 6, IEC; n = 9, SCC; n = 11), and (B) CD3^+^CD4^+^FoxP3^+^ T-cells to CD3^+^CD8^+^ T-cells (skin; n = 6, IEC; n = 7, SCC; n = 9). ns: not significant.

In order to assess whether there was an association between the proportion of CD4^+^T-cells or FoxP3^+^T-regs to CD8^+^ T-cells and SCC disease stage, we calculated the absolute numbers of each T-cell population within each sample and determined the ratio of CD4^+^T-cells to CD8^+^ T-cells (Fig. 5A) and the ratio of FoxP3^+^T-regs to CD8^+^ T-cells (Fig. 5B). We found that the ratio of CD4^+^T-cells to CD8^+^ T-cells was significantly higher in SCC when compared with IEC (*p*<0.05; Fig. 5A). However, the ratio of FoxP3^+^T-regs to CD8^+^ T-cells was not significantly different across the groups (*p* = 0.59; Fig. 5B). Taken together, the data suggest that there is a strong correlation between SCC diagnosis and an increased CD4^+^ T-cell to CD8^+^ T-cell ratio, which does not exist for an IEC diagnosis.

## Discussion

The identification of immune cells in SCC lesions has traditionally been performed using immunohistochemistry or immunofluorescence techniques. These techniques have the advantage of supplying the spatial location of immune cells within the lesion but the disadvantage that only a restricted number of markers can be simultaneously assessed [14]. In order to simultaneously identify multiple immune subpopulations therefore, we turned to multi-colour flow cytometry more commonly used to analyse cells that “crawl out” of cultured lesions [15], [16]. By enzymatically digesting fresh lesions and using stains to remove dead cells – a method shown previously to work well in mice [17], we show that it is possible to identify abundant cells like T-cells and investigate T-cell subpopulations to the required depth in the large majority of samples. The main advantage of this technique over the study of “crawl out” cells is that we were able to gain a consensus of the immune make-up of each sample that did not rely on the inherent motility of individual immune cell populations.

Using techniques such as immunohistochemical staining, it is well established that SCC lesions contain infiltrates of numerous immune cells including T-cells [14], [18], [19]. Several studies also report that numbers of FoxP3^+^ T-regs increase with progression to SCC [16], [20]. However, the correlation between T-cell infiltration as a whole and SCC disease progression is less clear, with separate groups showing SCC lesions to contain equivalent [14], reduced [21], or increased [16] numbers of T-cells when compared to AK, early AK or photo-damaged skin respectively. We show an increase in the percentage of T-cells present in IEC lesions when compared with photo-damaged skin (Fig. 3A), although there appeared to be no difference in the abundance of T-cells present in SCC compared with photo-damaged skin. The corresponding decrease in the percentage of cDC within IEC lesions (Fig. 3C) may be taken to suggest that an active T-cell response is occurring within IEC lesions, in which cDC migrate from the lesion to the draining lymph nodes, and consequently activated T-cells travel from the lymph nodes to the lesion. However, it is also possible that the large influx of T-cells into IEC made the percentages of other immune cells normally resident in skin, such as cDC, appear to decrease. We attempted to shed light on this possibility by weighing each sample prior to digestion to allow for comparisons of absolute numbers of each cell type between samples (to be expressed as cell count/gram of tissue), however we abandoned this approach when it became clear that there were large variations in fatty deposits between patient samples, leading to inaccurate estimations of actual *tumour* weight. Thus, the question of whether increased T-cell percentages in IEC correlate to increased T-cell activity will be further addressed in future studies through the analysis of T-cell activation markers like CD69.

Analysis of the NK population in IEC and SCC revealed that, while the percentage of NK cells was comparable between these two lesion types, both IEC and SCC appeared to show a decrease, albeit not statistically significant, in the percentage of NK cells present when compared with photo-damaged skin (Fig. 3B). Our observation that there may be a lower abundance of NK cells in SCC corresponds to previous findings in which the NK density within SCC lesions was reported to be approximately 10-fold lower than in the germinal centres of normal human tonsils [22]. In Head and Neck SCC, NK-mediated antibody-dependent cellular cytotoxicity (ADCC) has been linked to the efficacy of anti-EGFR monoclonal antibody therapies [23]. However, it remains to be determined whether there may be a correlation between relative NK abundance and response to anti-EGFR therapy in these patients.

Our data highlight the existence of important differences between skin, IEC, and SCC in the T-cell subpopulations that make up the total T-cell infiltrate. Notably, SCC appear to be infiltrated with a high proportion of CD4^+^ T-cells, which is in keeping with high proportions of these cells reported in perineoplastic infiltrates by immunohistochemistry [19], [24]. CD4^+^ T-cell infiltration, but not CD8^+^ T-cell infiltration, has been shown to correlate with the spontaneous regression of primary melanoma, BCC, keratoacanthoma, and a mouse model of UV-induced SCC [25], [26]. Given that precancerous IEC typically regress, while SCC do not, it is tempting to speculate that the properties of the CD4^+^ T-cells found in these lesions may differ. For example, a recent report described how an increase in so-called “chronically-stimulated” CD25^−^CD127^−^ CD4^+^ T-cells, but not conventional naïve (CD45RO^−^RA^+^CD27^+^CCR7^+^), effector (CD45RO^+^RA^±^CD27^−^CCR7^−^), or memory (CD45RO^+^RA^−^CD27^+^CCR7^+^) CD4^+^ T-cells, correlated with the regression of breast cancer during neoadjuvant chemotherapy [27]. Interestingly, we did not observe significant differences in the percentages of classical FoxP3^+^ T-regs between skin, IEC, and SCC. Therefore, the examination of other CD4^+^ T-cell subpopulations in precancerous lesions and SCC, which would be relatively straightforward using the 10-colour flow cytometry technique we have employed in this study, is the logical progression of this work. Additionally, and in light of our finding that the percentage of CD8^+^ T-cells within SCC lesions is lower than in IEC lesions, a similar analysis of CD8^+^ T-cell populations is also justified.

Further to the identification of decreased CD8^+^ T-cell numbers within SCC, an examination of the functionality of these cells becomes an important consideration. Recently, Bluth et al. observed that although phenotypically normal in terms of co-stimulatory marker expression, CD11c^+^HLA-DR^hi^ myeloid DC within SCC lesions are poor stimulators of allogeneic T-cells compared to DC derived from peritumoural non-lesional skin [28]. Furthermore, while treatment with a cocktail of maturation cytokines (IL-1β, IL-6, TNF-α, and PGE_2_) enhanced the stimulatory potential of DC from normal and peritumoural skin, DC derived from SCC lesions remained poor stimulators of T-cell proliferation [28]. Thus, it seems inherently possible that the function of other cells types within SCC, such as CD8^+^ T-cells, may also be suboptimal. Importantly, it remains to be determined whether the low CD8^+^ T-cell abundance in SCC may be linked to inherent defects in DC function within these lesions.

In this study we show that the CD4/CD8 T-cell ratio but not the T-reg/CD8 T-cell ratio is significantly higher in SCC when compared with IEC. In fact, the CD4/CD8 T-cell ratio was similar between photo-damaged skin and IEC, and may therefore represent an immunological diagnostic indicator of cutaneous SCC disease stage in immune competent patients. A significantly higher abundance of FoxP3^+^ T-reg with a correspondingly lower abundance of CD8^+^ T-cells has been described in SCC lesions from immune-suppressed kidney transplant recipients by immunohistochemistry [16], [29]. Indeed, higher numbers of T-regs in the blood of kidney transplant recipients was found to be predictive of SCC development, to be increased in those individuals with SCC, and to be significantly decreased following SCC resection, the latter being in contrast to post- Basal Cell Carcinoma (BCC) resection where T-reg numbers in the blood remained unchanged [30]. Whether a larger cohort of samples would reveal a similar T-reg/CD8 T-cell correlation in immune competent patients remains to be determined.

In addition to T-regs, the presence or absence of other cells with immunosuppressive potential within precancerous IEC and SCC lesions should also be considered. Although their numbers were small, we showed that iNKT cells are present in skin, IEC and SCC lesions. In mice, NKT cells have been demonstrated to be a regulatory cell population that plays a potent and critical role as suppressor cells in UV-irradiated skin, and have been shown to regulate the growth of UV-induced skin cancers [31], [32]. Recently, Mattarollo *et al.* identified that iNKT cells in mice are involved in the suppression of immune rejection to endogenously-expressed foreign-antigen in the skin [33], [34]. iNKT cells have the potential to be stimulatory or regulatory depending on the circumstances of their environment. IL-12 secretion by dendritic cells (DC) activates iNKT to secrete IFN-γ which in turn activates and/or induces the expansion of innate effector cells, including NK cells, neutrophils, DC and macrophages, as well as acquired effector cells such as CD4^+^ Th1 and CD8^+^ T-cells. However, when iNKT cells interact with IL-10-secreting DC they no longer produce IFN-γ, but instead produce IL-10, and thus exert suppressive effects [35]. To our knowledge, this study is the first to demonstrate the existence of iNKT within patient IEC and cutaneous SCC lesions, although assessing the function of these cells in these lesions may prove challenging as a consequence of their low abundance (<1% of the total CD45^+^ population).

Another immune cell type with immunosuppressive potential in the skin is the Mast cell (Bergot et al. manuscript under submission). Blocking Mast cell infiltration into the skin has recently been shown to significantly reduce the incidence of UV-induced immunosuppression and SCC development in a murine model [36]. While we did not specifically stain for Mast cells in this study, human Mast cells are reportedly both CD11b^−^ [37] and present in cutaneous SCC as determined through toluidine blue staining [38], and therefore may fit into the category of “other immune cells” in Figure 3F. Thus, a closer inspection of Mast cell function in IEC and SCC lesions seems timely.

This study demonstrates that while SCC display a high abundance of CD4^+^ T-cell infiltration, SCC have a lower abundance of CD8^+^ T-cells than IEC. The CD4/CD8 T-cell ratio may therefore represent an immunological diagnostic indicator of SCC over photo-damaged skin or precancerous stages of disease. The further analysis of CD4^+^ T-cell subpopulations and associated migration and activation stimuli within IEC and SCC lesions in particular thus warrants further clinical investigation.
